# Ecological and evolutionary implications of spatial heterogeneity during the off-season for a wild plant pathogen

**DOI:** 10.1111/nph.12646

**Published:** 2013-12-23

**Authors:** Ayco JM Tack, Anna-Liisa Laine

**Affiliations:** Metapopulation Research Group, Department of Biosciences, University of HelsinkiPO Box 65 (Viikinkaari 1), FI-00014, Helsinki, Finland

**Keywords:** epidemiology, genotype-by-environment interactions, host–parasite interactions, local adaptation, overwintering, plant–pathogen, spatial heterogeneity

## Abstract

- While recent studies have elucidated many of the factors driving parasite dynamics during the growing season, the ecological and evolutionary dynamics during the off-season (i.e. the period between growing seasons) remain largely unexplored.
- We combined large-scale surveys and detailed experiments to investigate the overwintering success of the specialist plant pathogen *Podosphaera plantaginis* on its patchily distributed host plant *Plantago lanceolata* in the Åland Islands.
- Twelve years of epidemiological data establish the off-season as a crucial stage in pathogen metapopulation dynamics, with *c*. 40% of the populations going extinct during the off-season. At the end of the growing season, we observed environmentally mediated variation in the production of resting structures, with major consequences for spring infection at spatial scales ranging from single individuals to populations within a metapopulation. Reciprocal transplant experiments further demonstrated that pathogen population of origin and overwintering site jointly shaped infection intensity in spring, with a weak signal of parasite adaptation to the local off-season environment.
- We conclude that environmentally mediated changes in the distribution and evolution of parasites during the off-season are crucial for our understanding of host–parasite dynamics, with applied implications for combating parasites and diseases in agriculture, wildlife and human disease systems.

## Introduction

While the question of why parasite populations crash has puzzled theoreticians and empiricists alike for nearly a century (Kermack & McKendrick, 1927), few studies have explored what happens during and after the cessation of growth and subsequent crash in size of parasite populations. Hence, we now know that in seasonal environments several mechanisms ranging from changing environmental conditions (Soper, 1929; Pascual & Dobson, 2005; Altizer *et al.*, 2006; Barrera *et al.*, 2012) and acquired immunity (Soper, 1929) to the evolution of increased host resistance (Duffy & Sivars-Becker, 2007) may contribute to the stabilization or decline of parasite populations, but we know hardly anything about pathogen dynamics during the ensuing off-season. In many systems where the host does not allow parasite growth or systemic (within-host) persistence during the off-season, the epidemic decline is matched by the production of specialized resting structures for survival (Combes, 2001; Agrios, 2005). Notably, the off-season may involve drought, heat and cold periods, depending on the climatic region and host and pathogen life history. By neglecting this off-season we may ignore a major part of the parasite puzzle: What role does the off-season play in explaining parasite population crashes and spatiotemporal disease dynamics? Do parasites exhibit genetic variation for survival in different off-season environments, and therefore show evolutionary responses to spatial and temporal heterogeneity during the off-season? Understanding this is nontrivial as off-season survival is a key determinant of the demographic and genetic structure of the epidemic in the next season.

The striking focus of the literature on the within-season growth of the parasite (often characterized by multiple generations of asexual reproduction; e.g. Tibayrenc & Ayala, 2012) has probably occurred for two reasons. First, parasitologists may have thought *a priori* that the off-season lacks any mechanistic underpinning or ecologically and evolutionarily interesting dynamics, and can hence be satisfactorily ignored, or described by a single stochastic parameter. Secondly, the off-season is notoriously hard to study, and even in well-studied pathosystems researchers may lack sufficient insight into the production, maturation, storage and revival of resting structures to allow for detailed experimentation. Possibly for these reasons, the majority of empirical and theoretical studies still regard off-season survival of parasites as a stochastic process or black box. Illustratively, the index of many ecological and evolutionary textbooks on host–parasite interactions entirely lacks an entry for ‘off-season survival’ or ‘overwintering’ (e.g. Thomas *et al.*, 2009; Schmid-Hempel, 2011). By contrast, the rapidly increasing research on the transmission phase of the parasite life cycle has revealed a wealth of information on spatiotemporal disease spread, and has elucidated a key role for genotype-by-environment interactions (Laine, 2008; Wolinska & King, 2009; Hall & Ebert, 2012).

**Fig 1:**
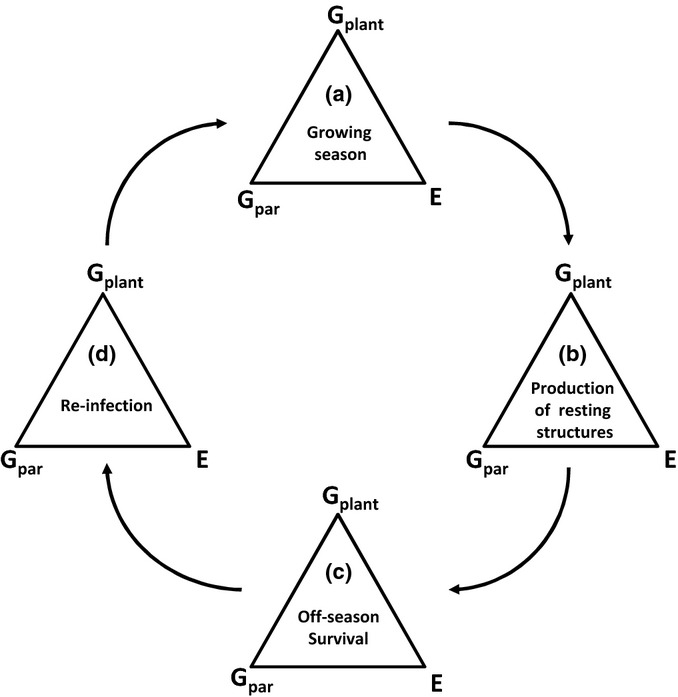
The majority of studies – and the classic disease triangle of phytopathology (McNew, 1960; Scholthof, 2007) – focus on the infection and epidemic stage of the parasite (triangle A and less frequently triangle D). As such, the disease triangle has emphasized for several decades how plant genotype (G_plant_), pathogen genotype (G_par_) and environment (E) jointly affect disease presence and intensity. However, we lack crucial insights into how genotype and environment interact during alternative life stages of parasites: production of resting structures (triangle B), off-season survival (triangle C) and infection of hosts at the start of the epidemic season (triangle D).

Survival during the off-season may – just like infection and epidemic spread during the growing season – be affected by the interaction between host genotype, parasite genotype and the environment (Fig. 1). However, while the impact of the abiotic and biotic environment on the off-season survival of parasites has been recognized for a long time (Roelfs, 1982; Marçais *et al.*, 1996; Bergot *et al.*, 2004), only recent studies have shown that parasite genotypes may differentially survive in the absence of the host, possibly through a trade-off with host transmission stages (Barrett *et al.*, 2011; Sommerhalder *et al.*, 2011). Further, to date we do not know whether parasite genotypes vary in their ability to survive different off-season environments. Given that spatiotemporal variation in environmental conditions is likely, it is crucial to assess whether parasites may adapt to changing off-season environmental conditions or shift distributions.

Off-season survival of parasites may depend on several life stages. First, parasite genotypes may vary in the production of resting structures (Fig. 1, triangle B). This variation may also be mediated by heterogeneity in the abiotic (e.g. temperature or humidity) or biotic (e.g. host genotype) environment in the period before the off-season (e.g. Gadoury & Pearson, 1988; Legler *et al.*, 2012; Asalf *et al.*, 2013). Secondly, the maturation and release of resting structures in spring may depend on the pathogen genotype, the genotype of the host on which the resting structures were produced, and environmental conditions during the off-season itself (Fig. 1, triangle C; Gadoury & Pearson, 1988; Abang *et al.*, 2006; Marçais *et al.*, 2009; Cohen *et al.*, 2013). Infection in spring may then depend on the spring conditions, pathogen genotype and receiving host genotype (Fig. 1, triangle D). In order to generalize our understanding of the impact of genotype and environment in shaping the spatial ecology and evolution of parasites during the off-season, we need to shift our focus to these multiple steps that underlie successful survival between growing seasons.

In this article, we investigate the off-season dynamics of the specialist obligate plant pathogen *Podosphaera plantaginis* on its perennial host *Plantago lanceolata*. In the Åland Islands in southwestern Finland, the pathogen population persists in a network of *c*. 4000 highly fragmented host populations, which range in size from a few square metres to several hectares, with a median size of 300 m^2^ (Nieminen *et al.*, 2004; Laine & Hanski, 2006; Laine, 2008; Tack *et al.*, 2013b) Yearly autumnal surveys conducted in the period 2001–2012 indicate that this highly dynamic pathogen metapopulation persists in the face of high population turnover, with approximately half of the pathogen populations going extinct from one year to the next (Laine & Hanski, 2006). Seasonality is considered to be a key driver of the metapopulation dynamics, with extinctions taking place during the environmentally harsh off-season, and colonizations occurring during the favourable growing season. Further modelling of the presence/absence pathogen data of *P. plantaginis* has predicted that there is spatial variation in overwintering success, with major consequences for local densities and the distribution of the pathogen during the following growing season (Soubeyrand *et al.*, 2009). However, as these studies were based on snapshot data collected every September, they may not accurately disentangle the extinctions during the off-season and the growing season. Here, we used data on the presence and local abundance of disease after infection had taken place in July for years 2011 and 2012. Furthermore, we investigated whether parasite genotype, environment and their interaction – which have been shown to have a major ecological and evolutionary impact on this system during the growing season (Laine, 2007, 2008; Tack *et al.*, 2013a) – play an equally important role during the off-season. More specifically, we (1) studied the extinction rate during the off-season using data from the end of one growing season and the beginning of the next year's growing season, and assessed whether environmental and spatial factors affect the extinction rate and abundance at the onset of disease dynamics; (2) assessed large-scale spatial and temporal variation in the production of resting structures of the powdery mildew across the metapopulation, and investigated whether environmental and spatial factors drive the observed variation; (3) demonstrated that resting structures are capable of infecting plants in spring; (4) conducted field experiments to test whether overwintering environment and genetic variation affect the ability of resting structures to infect plants in spring, and whether pathogens survive best in their sympatric (local) off-season environment; and (5) assessed the impact of resting spore production on small- and large-scale patterns in overwintering success in the pathogen metapopulation.

## Materials and Methods

### Study system

The powdery mildew *Podosphaera plantaginis* (Castagne; U. Braun & Takamatsu) is a fungal plant pathogen specific to *Plantago lanceolata* L. Like all members of the powdery mildews (Erysiphaceae), it is an obligate pathogen requiring living host tissue throughout its life cycle. During the growing season, *P. plantaginis* grows on the surface of the plant, with only its feeding roots (haustoria) penetrating the epidermis. Wind-dispersed spores (conidia) are produced on chains growing vertically on the leaf surface. Over the winter, *P. plantaginis* survives (see the Results section) through specialized resting structures (i.e. chasmothecia, formerly cleistothecia; Braun *et al.*, 2002). The resting structures of *P. plantaginis* are produced on the leaf surface (Supporting Information Fig. S1), and are relatively difficult to dislodge from the leaf (cf. Gadoury *et al.*, 2010). The number of resting structures on an infected leaf in autumn can vary strongly, ranging from no or few resting structures up to several thousand (e.g. *x*-axis in Fig. 5a). During development, the resting structures change from inconspicuous white, yellow and green to conspicuous brown and black (Fig. S1b). Each resting structure contains a single ascus, in which usually eight ascospores develop during successful maturation (Fig. S1c). Maturation takes place from late autumn to spring. During spring, the resting structures burst open and the ascospores can infect living plant material. Dormancy of resting structures for more than a single season, as equivalent to seed banks for plants, has not been reported and is regarded as unlikely for powdery mildews (Spencer, 1978; Bélanger *et al.*, 2002). A recent study has revealed that resting structures can be produced through haploid selfing in *P. plantaginis*, in contrast to the heterothallic nature of the majority of powdery mildews (Tollenaere & Laine, 2013).

The host plant *P. lanceolata* (ribwort plantain) is a monoecious, rosette-forming perennial herb (Sagar & Harper, 1964). The pollen is wind-dispersed and *P. lanceolata* is an obligate outcrosser (Ross, 1973). Seeds frequently drop close to the mother plant (Bos, 1992). In contrast to the pathogen, the spatial distribution of plant populations is consistent from year to year (Nieminen *et al.*, 2004; Ojanen *et al.*, 2013), possibly as a consequence of a combination of long-term seed bank and clonal propagation. During the winter in Åland the plant dies back to the rootstock and the resting structures remain attached to the dead leaves.

In the Åland archipelago in southwestern Finland, *P. lanceolata* populations are fragmented into discrete patches. The locations of the *c*. 4000 plant populations have been mapped since the early 1990s in the context of the Glanville fritillary butterfly (*Melitaea cinxia*) metapopulation project (Nieminen *et al.*, 2004; Ojanen *et al.*, 2013). The host plant mainly grows on dry meadows, pastures, and comparable habitats, which occur mostly as well-defined, discrete habitat patches (Nieminen *et al.*, 2004; Ojanen *et al.*, 2013). Since 2001, the incidence of *P. plantaginis* in this meadow network has been recorded systematically in early September of each year (Laine & Hanski, 2006). At this time, the fungus is highly conspicuous and resting structures become frequent (Fig. S1a). At the same time, several environmental and spatial variables have been recorded for each patch, including host plant coverage, distance to shore, road presence, host plant spatial connectivity, plant dryness and patch shadow (Nieminen *et al.*, 2004; Laine & Hanski, 2006; Ojanen *et al.*, 2013). Patch shadow was estimated in 2001 using a categorical scale: 1, no shadow; the entire patch is directly exposed to daylight for the majority of the day; 2, partly shadowed; a large part of the patch is shaded for part of the day; 3, shadowed; the entire patch is in shadow for most of the day. Light, temperature, and humidity are considered the key abiotic variables affecting disease establishment and development in powdery mildews, and some variation in these conditions is expected to be captured by this variable. Our focal populations in the Åland Islands are distributed across 14 geographical districts, and we use this division to obtain approximately equally sized units to visualize the extent of large-scale variation in overwintering success.

For more details on the methodology of the large-scale survey and the variables measured, we refer to Laine & Hanski (2006) and Ojanen *et al*. (2013).

### The key role of the off-season in pathogen population extinction

**Fig 2:**
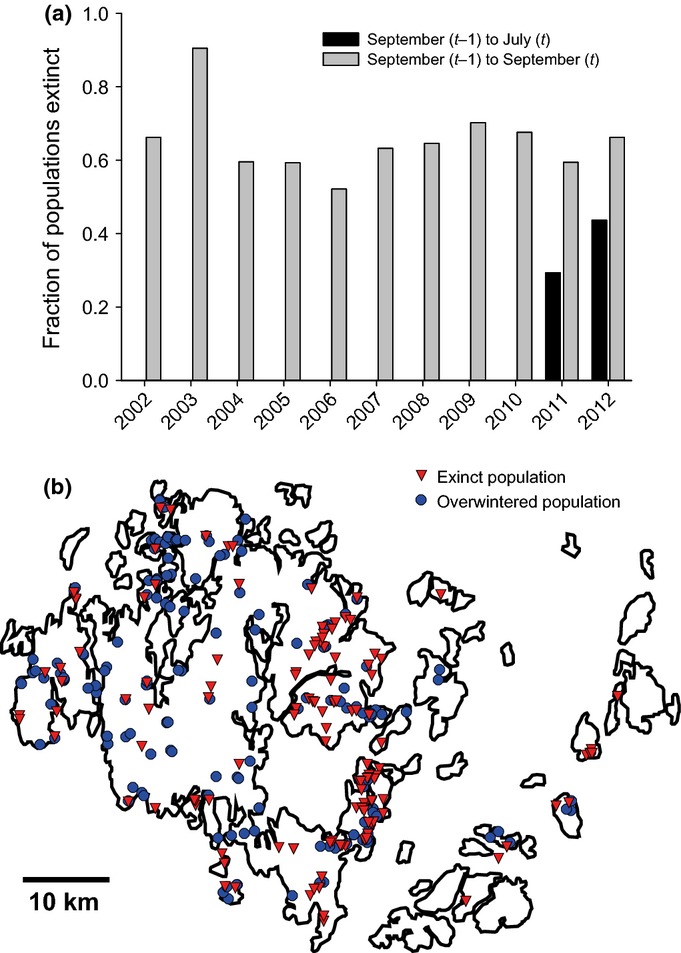
Extinction dynamics of the pathogen *Podosphaera plantaginis* in the *c*. 4000 host populations of *Plantago lanceolata* on the Åland Islands, southwestern Finland. (a) Grey bars represent the extinction rate estimated from the large-scale survey conducted each September since 2001 for the presence/absence of the pathogen *P. plantaginis*, calculated as the fraction of populations occupied in September of year *t* − 1 that were unoccupied in September of year *t*. The black bars represent the extinction rate estimated from more recent biannual surveys in September in year *t* − 1 and July in year *t*. (b) Data based on a survey in July 2012 of pathogen populations that were infected during the previous autumn, showing the spatial distribution of population survival in 2012 across the Åland Islands (Finland). Red triangles and blue circles refer to extinct and persisting populations, respectively.

While the long-term survey has indicated a high extinction rate of *P. plantaginis* from one year to the next (grey bars in Fig. 2a), such annual surveys cannot unambiguously disentangle extinctions during the off-season and the subsequent growing season. To accurately quantify the extinction rate during the off-season, we re-visited in early July 2011 a random subset of 47 populations occupied by the pathogen in the previous autumn. As this survey indicated that extinctions during the off-season were frequent (see Results), we visited the majority of populations occupied in September 2011 (*n *= 287 populations) again in July 2012 to further investigate the impact of spatial and environmental factors on off-season extinction and July abundance (triangles B–D, Fig. 1; Table 1). We chose to re-visit populations in early July, as this period is after the re-establishment of the powdery mildew from resting structures but well before epidemic spread of the pathogen within populations and across the metapopulation (R. Alanen, unpublished). In July 2011, when infection levels were low, we recorded for each visited population the presence/absence of the powdery mildew in the plant patch; at the same time, we scored local abundance of the powdery mildew as the total number of infected plants within the plant population (where the search was terminated when a threshold number of ten infected plants was detected; given low infection numbers in 2011, this only applied to 14.9% of the populations). In July 2012, when the overall pathogen abundance was much higher than in the previous year, local abundance was measured following a categorical scale: 0, absence of mildew; 1, 1–9 infected plants; 2, 10–99 infected plants; 3, 100–999 infected plants; and 4, 1000 or more infected plants.

**Table 1:** A summary of the experimental and observational materials used, the disease triangles addressed, and the models fitted for analyses

Interaction targeted	Triangle(s) addressed (Fig. 1)	Response(s) examined	Fixed effects (triangle)	Random effects (triangle)	Link^1^
(1) The key role of the off-season in pathogen population extinction	B–D	(a) Extinction (b) July abundance	*Environmental and spatial covariates*^2^		(a) Logit (b) Identity
(2) Spatial and temporal variation in the production of resting structures	B	Proportion of infected leaves with sexual resting structures^3^	*Environmental and spatial covariates*^2^		Logit
(3) Experiment on overwintering and spring infection	B–D	(a) Infection (0/1) (b) Proportion of infected leaves	*Number of resting structures* (B)	Pathogen population of origin (C) + overwintering site (C) + pathogen population of origin (C) × overwintering site (C) + micro-site (overwintering site) (C) + plant individual (pathogen population of origin) (C) + receiving plant genotype (D)	Logit
(4) The impact of resting structures on overwintering in the field at two spatial scales	B	Plant level: (a) Infection (0/1) (b) Proportion of infected leaves	*Number of leaves with resting structures*	Pathogen population	Logit
	B	Population level^4^: (a) Infection (0/1) (b) Number of infected plants	*Fraction of infected leaves with resting structures*		(a) Logit (b) Log

1 For continuous data, we assumed a normal distribution with an identity link; for count data, we assumed a Poisson distribution with a log link; for binomial data we assumed a binomial distribution with a logit link. Independent continuous variables are identified in italics.

2 Environmental and spatial covariates included are distance to shore, plant dryness, patch shadow, habitat openness, July rainfall, August rainfall, population age, host plant coverage, road presence and host plant spatial connectivity.

3 Separate models were constructed for 2010, 2011 and 2012.

4 Separate models were constructed for 2011 and 2012.

To investigate the temporal consistency of disease distribution within patches before and after the off-season, we additionally recorded the location of up to five infected plants in 259 host populations in September 2011. The next July, we surveyed the presence and absence of infection in these marked locations (1-m^2^ quadrats) as well as in an equal number of control quadrats at random locations. Random quadrats were selected to match the range of host density found in the marked quadrats to avoid a confounding effect of host density.

To analyse the impact of environmental and spatial factors on the spatial pattern of winter extinction and July abundance, we fitted a Bayesian spatial model using the integrated nested Laplace approximation (Cameletti *et al.*, 2012) as implemented in the package inla (Rue *et al.*, 2009; Lindgren *et al.*, 2011) in R version 2.15.1 (R Core Team, 2012). The advantage of this method is that it efficiently and accurately estimates both covariates and the spatial range of autocorrelation (as based on the Euclidean distance between populations). We included the environmental variables distance to shore, plant dryness, patch shadow, habitat openness, July rainfall, August rainfall and population age (i.e. how many years ago the pathogen population had been established by colonization, with a maximum value of 5) and the spatial factors host plant coverage, road presence and host plant spatial connectivity as explanatory covariates (Table 1). The average rainfall in July and August was estimated separately for each population using detailed radar-measured rainfall data. For further details on the statistical models, see Methods S1.

### Spatial and temporal variation in the production of resting structures

**Fig 3:**
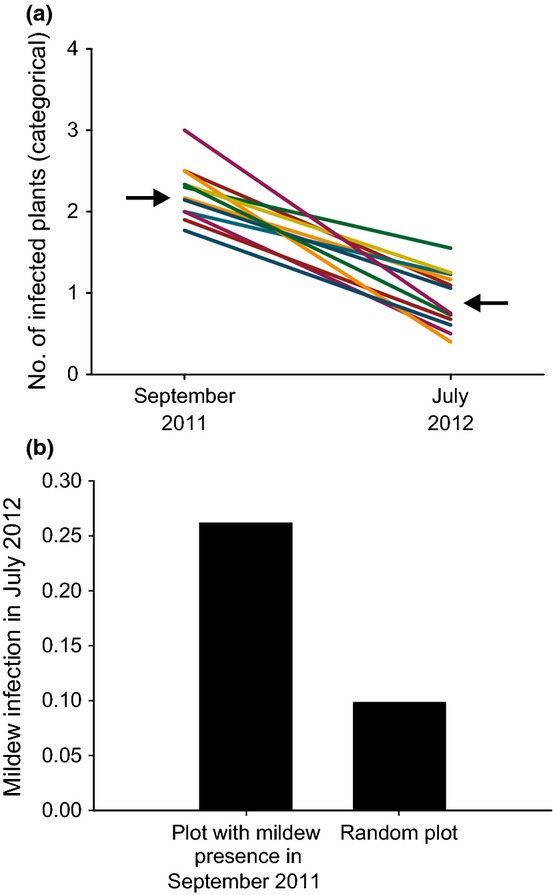
Patterns in off-season survival of the powdery mildew *Podosphaera plantaginis*. (a) The decrease in abundance during the winter for each of 12 geographical areas (‘districts’) within the Åland Islands, where arrows indicate the mean values. Note that the *y*-axis follows a categorical scale: 0, absence of mildew; 1, 1–9 infected plants; 2, 10–99 infected plants; 3, 100–999 infected plants; 4, 1000 or more infected plants. (b) Patterns of July infection within pathogen populations. Plots (1 m^2^) with known infection in the previous autumn (2011) have a likelihood of infection in July (2012) of 26.2%. Randomly selected plots have a much lower likelihood of infection (9.9%).

The last few decades have seen a rise in the number of studies that have shown that resting structures play a major role in the overwintering of powdery mildews, although experimental demonstrations in wild systems are still rare (Pearson & Gadoury, 1987; Braun, 1995; Mmbaga, 2000; Marçais *et al.*, 2009). To assess the extent and environmental causes of variation in the production of resting structures (Fig. 1, triangle B; Table 1), we surveyed natural variation in the production of resting spores across the metapopulation during the years 2010–2012 in the Åland Islands. In September 2010 and 2011, we selected a random subset of pathogen-infected plant populations for our survey (*n *= 91 out of 172 populations in 2010; *n *= 96 out of 268 populations in 2011), and nearly all populations were surveyed in September 2012 (*n *= 46 populations). For each infected plant population, we randomly selected approximately five infected plants (mean ± SD: 5.10 ± 1.57), or fewer when fewer than five infected plants were detected. For each plant we recorded whether infection and full-grown resting structures were present on ten haphazardly selected leaves (or fewer on small plants). Selecting five plants but scoring a large number of leaves allows for a similar survey effort across years and patches in the face of high temporal and spatial variation in the number of infected plants per population (cf. Fig. 3a).

To analyse the impact of environmental and spatial factors on the spatial pattern of resting structure production, we fitted a Bayesian spatial model using the integrated nested Laplace approximation as described in the previous section (using the same environmental and spatial covariates; Table 1).

### Experiment on overwintering and spring infection

We first carried out a trial overwintering experiment to investigate whether resting structures are able to infect plants in spring in this pathosystem, and to assess the impact of winter conditions (both indoors and outdoors) on the survival of resting structures. This experiment is described in detail in Notes S1.

Given successful infection in the trial experiment (Notes S1, Fig. S5), we set up a larger experiment in spring 2012 to pinpoint the impact of pathogen population of origin, overwintering site, and their interaction on the ability of resting structures to infect plants in spring (Fig. 1, triangles C and D; Table 1). In autumn 2011, we collected ten leaves bearing resting structures from five plants in five populations (pink circles in Fig. S2). These leaves were stored in polyester pollination bags (PBS International, Scarborough, UK) in a fully reciprocal design within these same five populations. Within each population, we stored the infected leaves in two locations (henceforth referred to as ‘micro-sites’) to probe the impact of micro-environmental variation within a single pathogen population. As a single leaf and population can contain multiple pathogen genotypes (Tollenaere *et al.*, 2012), we note that such an experiment cannot measure variation among individual pathogen genotypes for overwintering, but it may indicate whether there is genetic differentiation among pathogen populations (Tack *et al.*, 2012). After snow-melt in mid-April 2012, samples were recovered from the field (samples from a single micro-site were lost). To assess whether leaves bearing resting structures were able to infect plants in spring, individual leaves were positioned above a randomly assigned plant individual using two vertical sticks and horizontal iron wire. Plants were individually caged using a polyester pollination bag (10-1; PBS International; 1-window; 255 × 510 mm), as previous work has shown that infection develops well in these bags and spores cannot leave or enter (Laine, 2011; Notes S1). To reduce the impact of the receiving host plant genotype on the infection process, we used plant offspring from eight crosses between plant genotypes that were highly susceptible to a large number of pathogen genotypes (H. Susi, unpublished). Plants were scored on 19 June for the presence of powdery mildew infection. We used a total of 240 cages with 21 control cages in a largely balanced design. None of the control cages became infected. The experiment was conducted outdoors at Kumpula Botanic Garden (Helsinki, Finland).

To analyse the impact of pathogen population of origin, site of overwintering and their interaction on spring infection, we used the framework of generalized linear mixed-effects models (Table 1; Littell *et al.*, 2006). All models were fitted with the procedure GLIMMIX in sas 9.3 (Cary, NC, USA). For models with multiple interactions, we used the principle of backwards stepwise model simplification to arrive at a minimum adequate model, where variables were retained when *P *< 0.1 (Crawley, 2007). We used two different analyses to probe for the existence of local adaptation of pathogen resting structures to survival in their sympatric (local) off-season environment. See Methods S1 for a more detailed verbal description of the statistics and measures of local adaptation.

### The impact of resting structures on overwintering in the field at two spatial scales

We next aimed to investigate how natural variation in the amount of resting structures produced affects overwintering in the field at two spatial scales: individual plants and populations.

To assess the ability of resting structures to re-infect the plant on which they were produced (i.e. autoinfection), we enclosed 59 individual plants with variable numbers of resting structures inside polyester pollination bags at the end of the 2011 growing season. Placing them in these bags ensured that any infection visible on the plants in the following July resulted from the previous infection on the plant rather than from external sources of inoculum. As overwintering success may vary among plant populations, we selected ten plants in each of six populations (Fig. S2; *n *= 9 in one population). We also enclosed an additional seven uninfected control plants (*n *= 3 and *n *= 4 in two populations, respectively). In autumn, we recorded for each enclosed plant the total number of leaves with resting structures. Field cages were checked for infection in early July 2012. During this re-survey, nine cages did not contain a plant individual (because of mortality or dormancy) and one cage could not be recovered. None of the control cages became infected.

To assess the impact of resting structures on overwintering at the population level, we used data on the subset of populations where we collected both the July presence and abundance of the powdery mildew (see section ‘The key role of the off-season in pathogen population extinction’) and the level of resting structures in the previous autumn (see section ‘Spatial and temporal variation in the production of resting structures’). Data were available for 34 populations for the overwintering period from September 2010 to July 2011 and 89 populations from September 2011 to July 2012.

To assess the impact of resting structures on overwintering success at the plant level, infection (0/1) and disease intensity (number of infected leaves/total number of leaves) in July were modelled as a function of the number of leaves with resting structures in the previous autumn (Table 1). The pathogen population was used as a random factor to account for variation among populations in overwintering success. To investigate the impact of resting structures on overwintering at the population level, off-season survival and July abundance were modelled as a function of the fraction of infected leaves with resting structures in the previous autumn (Table 1).

## Results

### The key role of the off-season in pathogen population extinction

More than 30% of the pathogen populations went extinct during the winters of 2010/2011 and 2011/2012 (Fig. 2), thereby confirming that extinctions during the off-season play a major role in the high turnover rate in the pathogen metapopulation. Nonetheless, the extinction rates were in both years lower based on September–July surveys (*c*. 37%) than for the extinction rates based on the September–September surveys (*c*. 63%). Hence, while the off-season clearly represented a major period of extinctions (black bars), these data suggest that a significant number of extinctions also take place during the growing season (difference between black and grey bars in Fig. 2). Abundance estimates show that the abundance within powdery mildew populations crashed severely during the winter in each of the 12 geographical districts (Fig. 3a). Moreover, these data indicated that powdery mildew populations declined more severely in some districts than in others (repeated-measures ANOVA: *F*_11,253_ = 1.81 and *P* = 0.05). While a decline in densities during the off-season is not trivial given the large number of resting structures that can be produced even on a single leaf in autumn (cf. *x*-axis in Fig. 5a), only five out of 89 populations had higher densities in July than in the previous autumn and 20 populations remained at similar densities. This suggests that a decline (or crash) during the off-season is not universal but may be regarded as a general rule. At a finer spatial scale, disease disappeared during the off-season from 73.8% of the 1-m^2^ quadrats known to be infected during the previous autumn. Pathogen distribution within the patch was clearly related to the distribution of the disease in the previous autumn, as disease was more than twice as common in quadrats with known disease incidence in the previous autumn as compared to random quadrats (Fig. 3b; ANOVA: *F*_1,1290_ = 18.43 and *P *< 0.001).

Our Bayesian model of the survey data in July 2012 revealed that pathogen extinction was high in patches that were: small (in terms of host coverage); exhibited no dryness of plants in the previous autumn; received little rainfall in the previous July; and were recently colonized (Table S1). When populations survived during the off-season, the abundance in July was positively affected by plant coverage, and negatively affected by distance to shore (Table S2). The mode for the spatial range (reflecting the range of spatial autocorrelation) was 6.7 and 7.9 km for extinction and abundance, respectively (Fig. S3).

### Spatial and temporal variation in the production of resting structures

**Fig 4:**
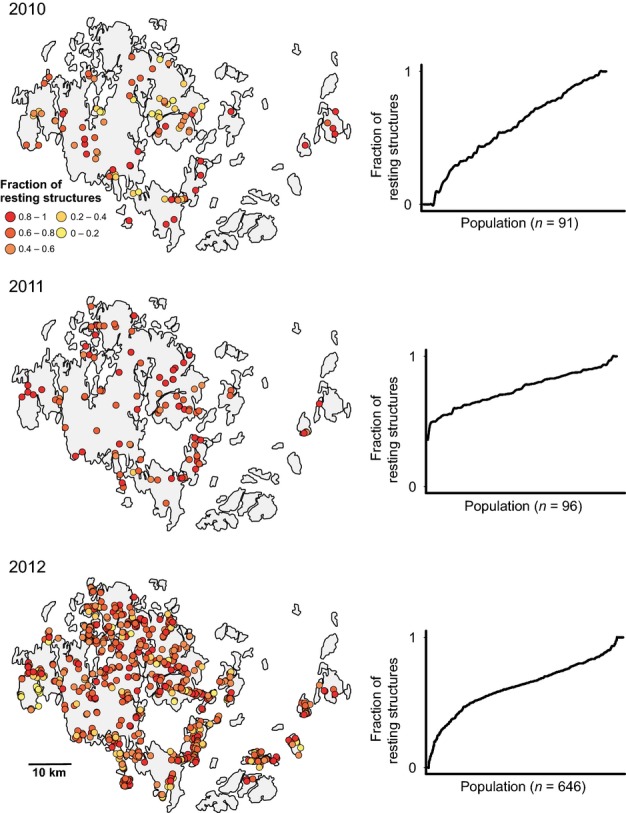
Spatial variation in the production of resting structures (i.e. fraction of infected leaves with resting structures) by the powdery mildew *Podosphaera plantaginis* for each of three years. The graphs on the right show the distribution of the fraction of infected leaves with resting structures across populations. For each year, the fraction of infected leaves with resting structures is highly variable among populations, but particularly so in 2010 and 2012.

The production of resting structures was highly variable among populations (Fig. 4) and affected by several environmental factors in each of the years 2010–2012, though the impact of individual factors varied strongly among years (Table S3). In 2010, the formation of resting structures increased with plant dryness and decreased with rainfall in August (Table S3). In 2011, August rainfall likewise decreased the formation of resting structures, whereas July rainfall had the opposite effect, indicating the complex impact of rainfall across the growing season. Population age negatively affected the formation of resting structures in 2011, whereas it positively affected resting structures in 2012. Habitat openness had a strong positive impact on the formation of resting structures in both 2011 and 2012, while host spatial connectivity had a positive impact on the formation of resting structures in 2011 only. Production of resting structures was spatially correlated in each of the three years, with the mode for the spatial range varying from 2.0 to 4.6 km in 2010 and 2012, respectively (Fig. S4). Notably, while the fraction of infected leaves with resting structures covered the full range from zero to one in 2010 and 2012, there were no populations with no or few resting structures in 2011 (Fig. 4). The production of resting structures across populations was highly uncorrelated among years (all pairwise Pearson correlations *P* > 0.3).

### Experiment on overwintering and spring infection

**Fig 5:**
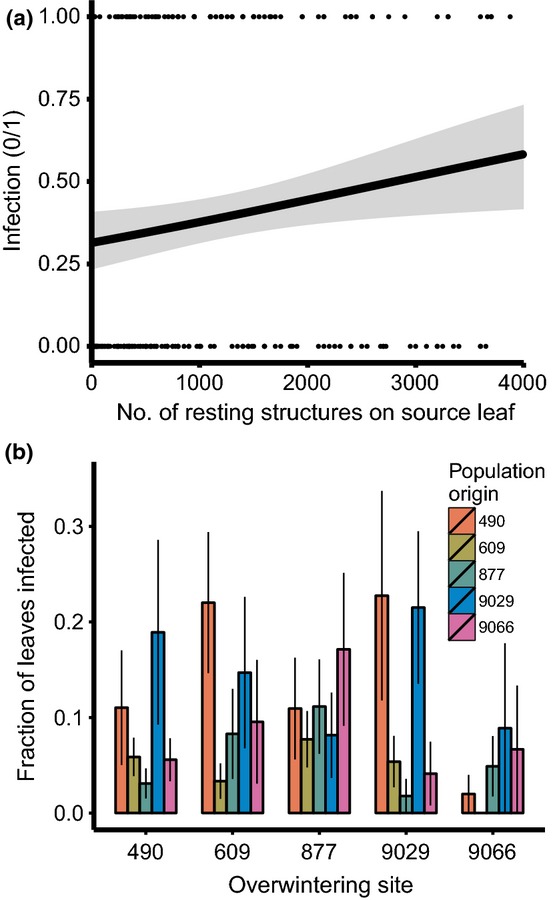
Some examples of factors that affect overwintering of the pathogen *Podosphaera plantaginis* in a reciprocal experiment. (a) The impact of the quantity of resting structures on spring infection (a single data points falls to the right of the plotted range). (b) The effect of the interaction between pathogen population of origin and overwintering site on infection intensity in spring. The black line and associated grey shaded area in (a) show the logistic regression line and its 95% confidence interval, respectively. In (b) are plotted empirical means ± SE.

The large overwintering experiment was aimed at disentangling the impact of pathogen population of origin and large- and small-scale environmental variation on the viability of resting structures and subsequent infection of plants in spring. As expected, both the infection (0/1) and disease prevalence (i.e. proportion of infected leaves) on caged plants in spring were positively correlated with the number of resting structures on the overwintered leaf (*F*_1,161_ = 4.71, *P* = 0.03 and *F*_1,137_ = 26.13, *P* < 0.001, respectively; Fig. 5(a); see Table S4 for detailed statistical results). Furthermore, the proportion of leaves infected was affected by the interaction between population of origin and the location of overwintering (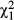 = 15.43; *P* < 0.001; Fig. 5b), suggesting that pathogen genotypes from different populations vary in their ability to survive a range of off-season environments. Variation among plant individuals within population of origin (e.g. attributable to within-population variation in pathogen genotype) as well as the location of the bags within an overwintering location (i.e. micro-environmental variation during the off-season) explained additional variation in the infection intensity (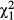 = 62.05, *P* < 0.001 and 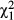 = 3.87, *P* = 0.05, respectively). Finally, despite the selection of generally susceptible plant genotypes as trap plants in the cages, we still detected variation in infection level among plant genotypes (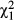 = 19.90; *P* < 0.001). We detected a trend for higher disease infection in sympatric (back-transformed least-squares mean ± SE: 0.064 ± 0.032) as compared to allopatric (back-transformed least-squares mean ± SE: 0.043 ± 0.021) combinations using two alternative modelling approaches (Methods S1), suggesting some support for local adaptation by the pathogen to its sympatric (local) overwintering environment (*F*_1,148_ = 4.66, *P* = 0.03 and *F*_1,133_ = 3.68, *P* = 0.06 for models 1 and 2, respectively).

### The impact of resting structures on overwintering in the field at two spatial scales

Finally, we investigated the impact of resting structures on overwintering at the level of individual plants and populations. At the level of the individual plant, a large fraction of the plants that were infected and enclosed in a cage during the off-season were infected during the following spring (i.e. ‘auto-infection’; 41% or 20 out of 49 plants). The control plants (*n* = 7) all remained without infection. There was a positive relationship between the number of leaves with resting structures in the previous autumn and infection and disease intensity in July (*F*_1,42_ = 2.21, *P* = 0.14 and *F*_1,42_ = 33.23, *P* < 0.001, respectively). There was significant variation among the pathogen populations in the number of infections and the disease intensity (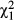 = 4.30, *P* = 0.02 and 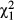 = 50.16, *P* < 0.001, respectively).

The number of infected plants in a population in 2011 was positively correlated with the fraction of infected leaves with resting structures in the previous autumn (*F*_1,32_ = 4.07; *P* = 0.05), but no significant effect was detected for the presence/absence of infection (*F*_1,32_ = 0.93; *P* = 0.34). The fraction of infected leaves with resting structures in September 2011 did not affect survival or infection intensity in July 2012 (*F*_1,87_ = 0.15, *P* = 0.70 and *F*_1,87_ = 0.06, *P* = 0.80, respectively).

## Discussion

Few previous studies have investigated the ecological and evolutionary dynamics of host and parasites during the off-season. In this study, we combined observational and experimental studies to demonstrate that: the ephemeral nature of local pathogen populations was directly related to the off-season, when 40% of the populations went extinct and local population abundances strongly declined across the metapopulation; environmental and spatial factors strongly affected pathogen overwintering and the production of resting structures; and pathogen population of origin and the off-season environment interacted to jointly shape infection intensity in spring, with a weak signal of pathogen adaptation to the local off-season environment. Overall, while an increasing number of studies have explored how genotype and environment shape parasite transmission and evolution during the growing season (triangle A in Fig. 1; e.g. Wolinska & King, 2009), the current study emphasizes that equally fascinating, complex and unexplored ecological and evolutionary dynamics play out across the off-season.

### The extended disease triangle

McNew's disease triangle (1960) focuses on how the environment, plant genotype and pathogen genotype jointly shape disease dynamics, with a clear focus on the growing season. Here, we extend the disease triangle to the off-season.

We detected a strong impact of environmental and spatial factors on overwinter survival and July abundance (triangles B–D, Fig. 1) and the production of resting structures (triangle B, Fig. 1). However, the identity of the spatial and environmental drivers varied among response variables and among years. Higher plant coverage (representing patch area) decreased extinction and increased July abundance, thereby re-emphasizing the important role of patch area in classic metapopulation dynamics (Hanski, 1994). Environmental factors such as a higher percentage of dried plants and higher July rainfall increased winter survival (probably through direct effects on pathogen growth), whereas August rainfall (−) and habitat openness (+) were the most consistent predictors of the presence of resting structures. Population age increased the likelihood of both off-season survival and the production of resting structures in 2012, probably because these patches provide a more optimal habitat to the pathogen. Despite the detection of several environmental and spatial variables, the spatial scale of autocorrelation (extending up to 8 km) suggests that our environmental and spatial variables fail to capture all spatial variation. This may not be surprising, particularly given the fact that the environmental variables included in the model were originally selected based on their potential relevance for the epidemiology during the growing season. Importantly, the scale of autocorrelation may guide us in the identification of environmental variables relevant for the off-season (e.g. snow cover). While rarely considered, spatial patterns in the pathogen genetic and phenotypic distribution may further explain patterns of spatial autocorrelation. For example, while the current study does not directly address the role of plant or pathogen genotype in the production of resting structures, other experimental work in this system shows that the production of resting structures by pathogens strains is differentially affected by light (Tollenaere & Laine, 2013) and soil environment (A. J. M. Tack, unpublished). Comparable studies in other systems reveal a key role for both host resistance and environment in the production of resting structures of grape powdery mildew *Erysiphe necator* (Gadoury & Pearson, 1988; Legler *et al.*, 2012) and the impact of pathogen genotype, temperature and their interaction on the production of resting structures of strawberry powdery mildew *Podosphaera aphanis* (Asalf *et al.*, 2013). Jointly, these observational and experimental studies suggest that both the environment and genotype may explain variation in the production of resting structures (triangle B, Fig. 1).

The maturation and viability of the resting structures may depend on pathogen genotype and environment experienced during the off-season (triangle C, Fig. 1). Here, our experiment revealed a relatively weak effect of pathogen population of origin or overwintering site *per se*: instead, pathogens from different locations responded differently to the same off-season environment (i.e. a genotype × environment interaction). While the effect of the off-season environment on the viability of resting spores is well known (Gadoury & Pearson, 1988; Cohen *et al.*, 2013), few studies have investigated the effect of pathogen origin or genotype × environment interactions. Finally, our experiment showed a significant impact of receiving plant genotype on infection in spring (Fig. 1, disease triangle D). While not the focus of our experiment, this warrants further study and suggests that plant genotype may be critical to safeguard spring infection. Such a notion is in line with results from this (Laine, 2005, 2008; Tack *et al.*, 2013b) and other (Thompson & Burdon, 1992; Laine *et al.*, 2011) disease systems demonstrating that plant genotype plays a key role in the infection process. Notably, the pattern of high temporal consistency of disease distribution within plant populations between autumn and July infections (Fig. 3b) may then be a result of (a combination of) dispersal limitation, micro-environmental variation and spatial variation in plant genotype (Tack *et al.*, 2013a).

While the fraction of leaves with resting structures in autumn 2010 explained disease intensity in July 2011, we detected no relationship between levels of resting spores in September 2011 and infection in July 2012. The absence of a pattern for the latter year may be explained by the absence of populations with no or few resting structures in September 2011 (Fig. 4). Interestingly, this relatively high fraction of resting structures in nearly every pathogen population in September 2011 may also explain the large overall increase in the pathogen metapopulation from September 2011 to September 2012 (from 268 to 633 pathogen populations, respectively).

### The evolutionary implications of the extended disease triangle

While the key aim of McNew's disease triangle was to understand and predict disease severity, the inclusion of genetic factors and the potential for genotype-by-environment interactions within the disease triangle provides a direct link between epidemiological patterns and evolutionary processes. Previous results in this pathosystem have indicated the key role for genotype-by-environment (temperature and nutrient levels) interactions in infection dynamics (Laine, 2007) and patterns of local adaptation (Laine, 2008). We now have accumulating evidence that genotype × environment interactions may also be important during the production of resting structures (triangle B, Fig. 1; Tollenaere & Laine, 2013) and during the ensuing off-season (triangle C, Fig. 1; Fig. 5). Such studies indicate the existence of differentiation among pathogen genotypes and pathogen populations in the ability to produce resting structures and subsequently survive a range of environmental conditions during the off-season. At the same time, our results show that the ecological and evolutionary drivers of interactions may vary in time, and the dynamics of an interaction can thus be a composite of temporally distributed genotype × environment (or, more speculatively, genotype × genotype × environment) interactions. The existence of temporal variation in (genotype ×) genotype × environment interactions has important implications for coevolution within metapopulations and across larger geographical scales for all forms of interactions between species (Thompson, 2005).

Pathogen population differentiation and its interaction with the off-season environment indicate that genetic variation exists for the pathogen to adapt to spatial variation in environmental conditions within the metapopulation. Our reciprocal transplant experiment revealed a marginally significant pattern of local adaptation (*P* = 0.06): while resting structures from four out of five populations resulted in relatively high infection intensity in spring when stored in their local population during the off-season, this pattern was reversed in population 609 (Fig. 5b). The absence of a consistent pattern of local adaptation during the off-season may be explained by at least three mechanisms. First, local adaptation may be swamped by high gene flow (Slatkin, 1987; Tack & Roslin, 2010) or wiped out by frequent extinctions. A classic study on the mummy berry fungus (*Monilinia vaccinii-corymbosi*) on blueberry (*Vaccinium* spp.) illustrates this pattern: while spring germination of overwintering structures was in some locations adapted to be synchronized with the phenology of bud break in the host cultivar, such adaptation was absent when early and late cultivars were grown in close proximity (Lehman & Oudemans, 2000). Secondly, there may be a trade-off between off-season survival and within-season growth, thereby counteracting selection in separate stages of the pathogen life cycle (cf. Carson, 1998; Martinez *et al.*, 2005; Abang *et al.*, 2006; Barrett *et al.*, 2011; Sommerhalder *et al.*, 2011). Thirdly, the magnitude of yearly variation in overwintering conditions may (far) exceed heterogeneity in summer conditions: while resting structures may be protected from degeneration by a thick and persistent snow cover in some years (cf. Pearson & Gadoury, 1987), the lack of a continuous snow cover in other years may expose the resting structures – at least in some populations – to extreme weather and subzero temperatures. Such a mosaic pattern of local adaptation has previously been reported in this study system for pathogen performance during the growing season (Laine, 2005, 2008). The existence of genetic variation in our study is in contrast with studies on the oak powdery mildew (*Erysiphe alphitoides*), which showed the absence of genetic variation in the timing of spore release (and therefore local adaptation) in spring (Marçais *et al.*, 2009; Desprez-Loustau *et al.*, 2010).

### Conclusions

We demonstrate that parasite survival during the off-season is crucial to our understanding of disease metapopulation dynamics and evolutionary responses of the pathogen to environmental heterogeneity. We expect that ecological and evolutionary changes during the largely unexplored off-season play a similar role in the dynamic behaviour of a wide range of other host–parasite systems, irrespective of whether the parasite survives as resting structures, in low densities on the few remaining or susceptible hosts, or saprophytically. Unravelling the ecological and evolutionary drivers behind the off-season dynamics across host–parasite systems, both empirically and theoretically, offers an exciting opportunity for future research and is needed to generate predictions regarding disease dynamics from one season to the next. From an applied perspective, insights into the off-season may be pivotal in combating diseases and parasites in agricultural, wildlife and human disease systems (Bartlett, 1957; Altizer *et al.*, 2006; Rambaut *et al.*, 2008; Gunning & Wearing, 2013; Jaspers *et al.*, 2013) and in the design of environmentally friendly means of managing diseases and parasites (Peterson *et al.*, 2005; Legler *et al.*, 2012; Cohen *et al.*, 2013).

## Supporting Information

Fig S1 Resting structures of the powdery mildew *Podosphaera plantaginis*. **Fig. S2** Locations of overwintering cages and locations where resting structures were collected in autumn and/or stored during the off-season. **Fig. S3** Posterior distribution of the spatial range estimate for a spatial Bayesian model for winter extinction of the pathogen *Podosphaera plantaginis* from 2011 to 2012 and pathogen abundance in July 2012. **Fig. S4** Posterior distribution of the spatial range estimate for the fraction of infected leaves with resting structures for the pathogen *Podosphaera plantaginis* in its host populations in Åland, southwestern Finland. **Fig. S5** Fraction of infected cage plants in the overwintering trial experiment. **Table S1** Factors that affect the extinction of the powdery mildew pathogen *Podosphaera plantaginis* during the off-season in its host populations in Åland, southwestern Finland **Table S2** Factors that affect the July abundance of the powdery mildew pathogen *Podosphaera plantaginis* in infected host populations in Åland, southwestern Finland **Table S3** Factors that affect the fraction of infected leaves with cleistothecia for the pathogen *Podosphaera plantaginis* in its host populations in Åland, southwestern Finland **Table S4** The impact of population of origin and overwintering site on the ability of resting structures to infect individually caged plants in spring **Methods S1** A detailed description of the statistical methods. **Notes S1** A trial experiment on overwintering survival using indoor and outdoor overwintering sites.
