# Supplementary material for: Ecological and evolutionary implications of spatial heterogeneity during the off-season for a wild plant pathogen
Source: New Phytol. 2013 Dec 23;202(1):297–308. doi: 10.1111/nph.12646 (PMC4285854; doi:10.1111/nph.12646)
Supplement: Fig S1 — Resting structures of the powdery mildew Podosphaera plantaginis. Fig. S2 Locations of overwintering cages and locations where resting structures were collected in autumn and/or stored during the off-season. Fig. S3 Posterior distribution of the spatial range estimate for a spatial Bayesian model for winter extinction of the pathogen Podosphaera plantaginis from 2011 to 2012 and pathogen abundance in July 2012. Fig. S4 Posterior distribution of the spatial range estimate for the fraction of infected leaves with resting structures for the pathogen Podosphaera plantaginis in its host populations in Åland, southwestern Finland. Fig. S5 Fraction of infected cage plants in the overwintering trial experiment. Table S1 Factors that affect the extinction of the powdery mildew pathogen Podosphaera plantaginis during the off-season in its host populations in Åland, southwestern Finland Table S2 Factors that affect the July abundance of the powdery mildew pathogen Podosphaera plantaginis in infected host populations in Åland, southwestern Finland Table S3 Factors that affect the fraction of infected leaves with cleistothecia for the pathogen Podosphaera plantaginis in its host populations in Åland, southwestern Finland Table S4 The impact of population of origin and overwintering site on the ability of resting structures to infect individually caged plants in spring Methods S1 A detailed description of the statistical methods. Notes S1 A trial experiment on overwintering survival using indoor and outdoor overwintering sites. [file nph0202-0297-SD1.pdf]

Table S1. Factors that affect the extinction of the powdery mildew pathogen *Podosphaera plantaginis* during the off-season (2011/2012) in its host populations in the Åland Islands, southwestern Finland. Shown are the results from a spatial Bayesian model using LAPLACE approximation. Estimates in bold are significant as based on a decrease in DIC. The spatial pattern of winter extinction is shown in Fig. 2b in the main manuscript.

Factor	mean	0.025quant	0.5quant	0.975quant	DIC
(Intercept)	1.1185	0.0595	1.1390	2.0728	
**Plant coverage (m^2)**	**-0.0180**	-0.0380	-0.0173	-0.0018	246.31
**Plant dryness (%)**	**-0.0722**	-0.1290	-0.0705	-0.0248	247.37
**July rainfall (mm)**	**-0.0222**	-0.0406	-0.0225	-0.0021	253.52
**Population age (yr)**	**-0.3331**	-0.7259	-0.3248	0.0136	245.39
Full model					243.95

Table S2. Factors that affect the July abundance (year 2012) of the powdery mildew pathogen *Podosphaera plantaginis* in infected host populations in the Åland Islands, southwestern Finland. Shown are the results from a spatial Bayesian model using LAPLACE approximation. Estimates in bold are significant as based on a decrease in DIC.

Factor	mean	0.025quant	0.5quant	0.975quant	DIC
(Intercept)	1.7342	1.2615	1.7564	2.0721	
**Plant coverage (m^2)**	**0.0030**	0.0013	0.0030	0.0048	222.82
**Distance to shore (m)**	**-0.0001**	-0.0003	-0.0001	0.0000	216.41
Spatial connectivity	-0.0045	-0.0118	-0.0046	0.0035	214.57
Full model					215.63

Table S3. Factors that affect the fraction of infected leaves with resting structures of the pathogen *Podosphaera plantaginis* in its host populations in the Åland Islands, southwestern Finland. Shown are the results from year-specific spatial Bayesian models using LAPLACE approximation. Estimates in bold are significant as based on a decrease in DIC. The spatial pattern of resting spore production is shown in Fig. 4 in the main manuscript.

2010

Factor	mean	0.025quant	0.5quant	0.975quant	DIC
(Intercept)	0.8689	0.5444	0.8722	1.1744	
**Plant dryness (%)**	**0.0042**	0.0010	0.0042	0.0075	-114.46
**August rainfall (mm)**	**-0.0050**	-0.0093	-0.0051	-0.0005	-77.99
Full model					-388.90

2011

Factor	mean	0.025quant	0.5quant	0.975quant	DIC
(Intercept)	0.3838	0.1695	0.3837	0.5988	
**Habitat openness (0/1)**	**0.3681**	0.2048	0.3680	0.5321	-99.23
**Spatial connectivity**	**0.0021**	0.0004	0.0021	0.0039	-121.72
**July rainfall (mm)**	**0.0020**	0.0003	0.0020	0.0036	-114.15
**August rainfall (mm)**	**-0.0029**	-0.0051	-0.0029	-0.0006	-125.35
**Population age (yr)**	**-0.0238**	-0.0378	-0.0238	-0.0097	-105.98
Full model					-139.30

2012

**Factor**	**mean**	**0.025quant**	**0.5quant**	**0.975quant**	**DIC**
(Intercept)	0.3351	0.2515	0.3351	0.4183	
Plant coverage (m^2)	0.0000	0.0000	0.0000	0.0000	-141.38
**Habitat openness (0/1)**	**0.0004**	-0.0012	0.0004	0.0021	-134.29
**Population age (yr)**	**0.0001**	-0.0013	0.0001	0.0015	-137.84
Full model					-139.85

Table S4. The impact of population of origin and off-season storage location on the ability of resting structures to infect individually caged plants in spring. See Fig. 5 of the main manuscript for a visual depiction of the data.

**Factor**	**Infection**	**Infected leaves / Total number of leaves**
Pathogen population of origin	X^2=0.00, p=1.00	X^2 = 0.38, p = 0.54
Storage location	X^2=0.00, p=1.00	X^2=0.00, p=1.00
Pathogen population of origin x *Storage location*		X^2=15.43, p<0.001
Micro-site (Storage location)	X^2=3.66, p=0.06	X^2=3.87, p=0.05
Plant (Pathogen population of origin)	X^2=2.83, p=0.09	X^2=62.05, p<0.001
Receiving plant genotype		X^2=19.90, p<0.001
Number of resting structures	$F_{1,161}$ = 4.71; p=0.03	$F_{1,137}$=26.13, p<0.001

Fig. S1. Resting structures of the powdery mildew *Podosphaera plantaginis*. Panel A shows the conspicuous brown/black spores as detected in field surveys. Panel B depicts the mature (brown) and immature (white/green) resting structures as seen under a stereomicroscope. Panel C shows a resting structure revealing its single ascus with up to eight ascospores. Photo credits: Anna-Liisa Laine and Riikka Alanen.

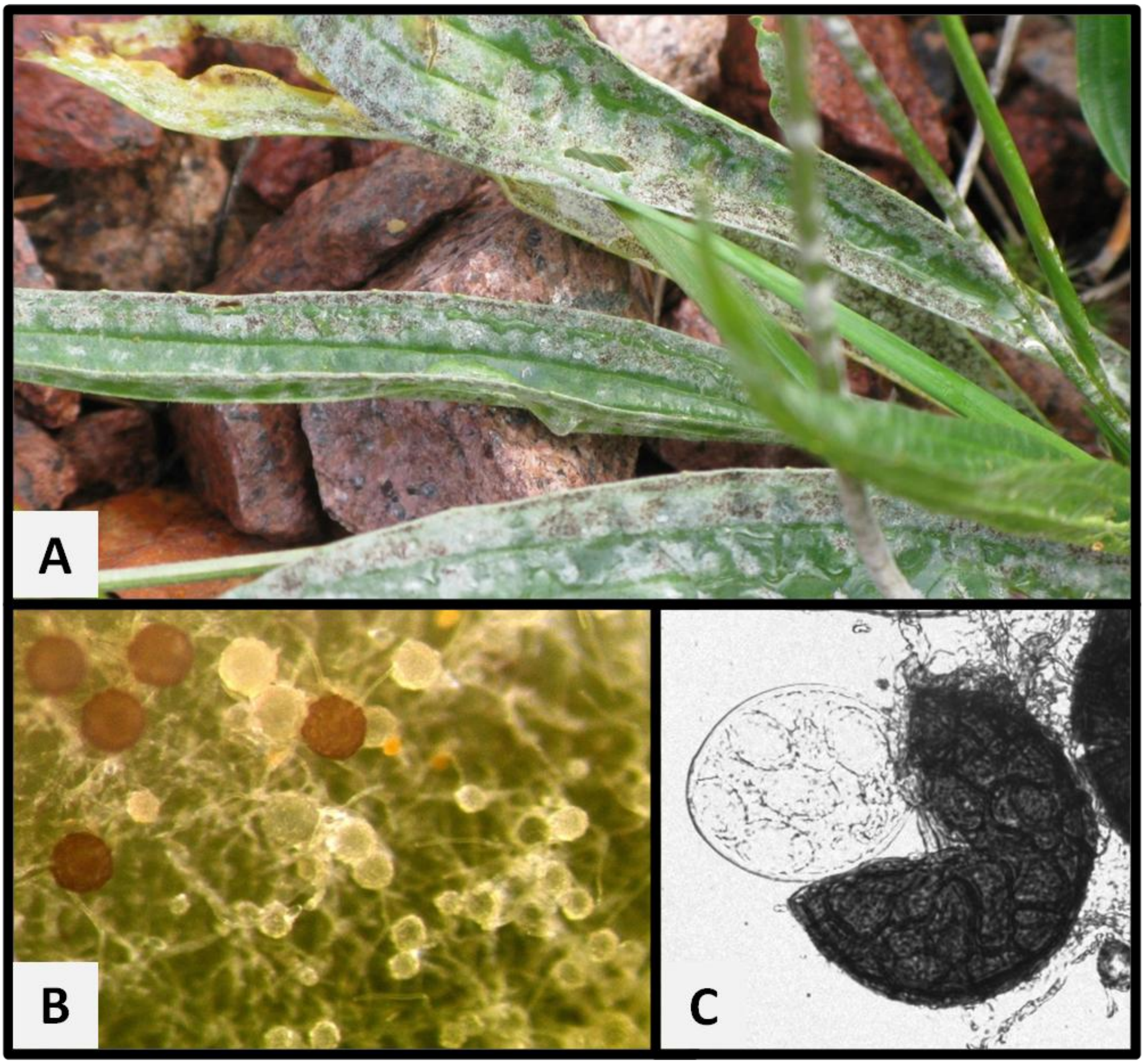

Fig. S2. Locations where resting structures were collected in autumn and/or stored during the off-season. Red triangles indicate pathogen populations where resting structures were collected in autumn 2010 and stored under various indoor conditions. Green squares indicate populations where resting structures were collected in autumn 2010, which were subsequently stored during the off-season in both the green and purple squares. The pink circles show the location where resting structures were collected in autumn 2011, and subsequently stored reciprocally in these same five locations. In autumn 2011, we caged *c.* ten plants in each of six populations (pink and yellow circles).

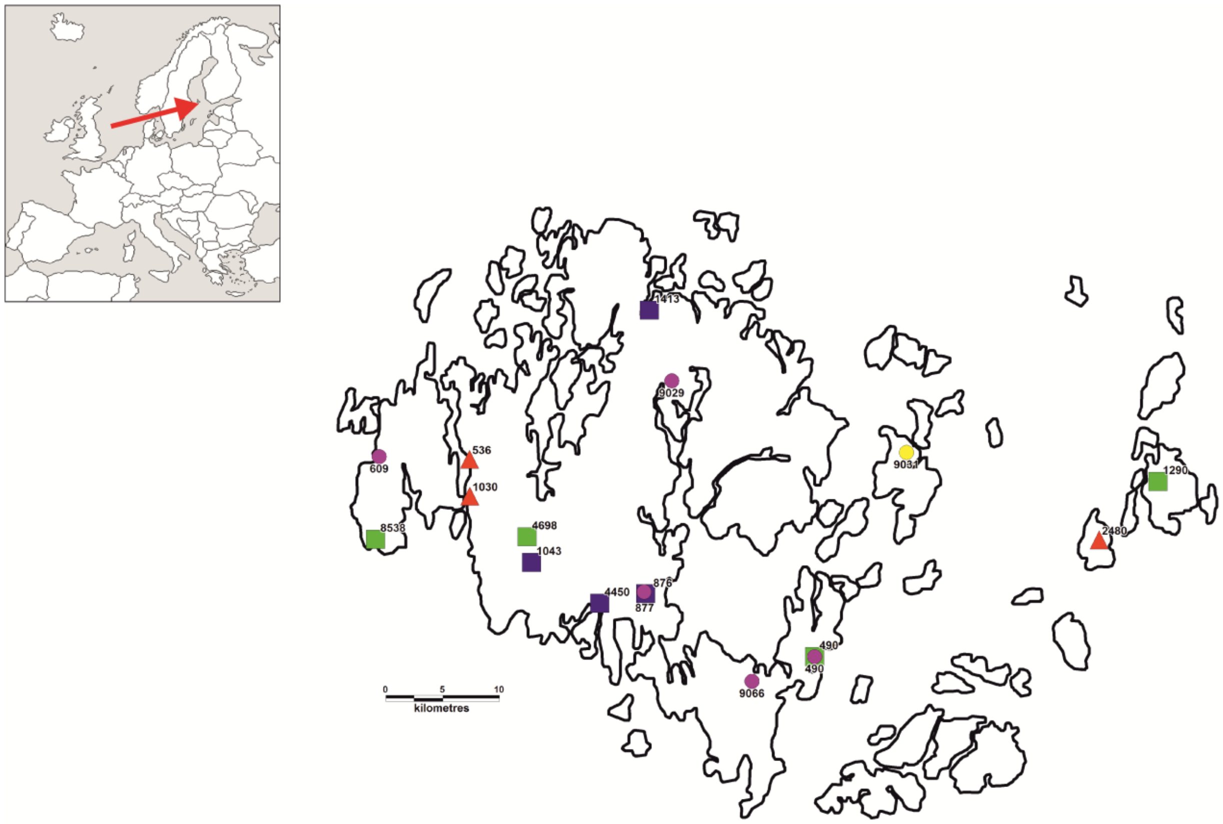

Fig. S3. Posterior distribution of the spatial range estimate for a spatial Bayesian model for A) winter extinction of the pathogen *Podosphaera plantaginis* from 2011 to 2012 and B) pathogen abundance in July 2012. The spatial pattern of winter extinction is shown in Fig. 2b in the main manuscript.

A)

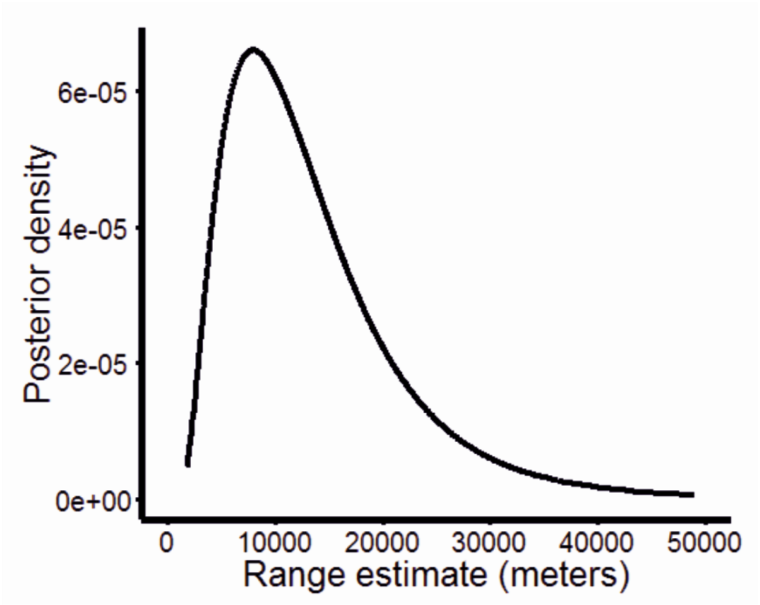

B)

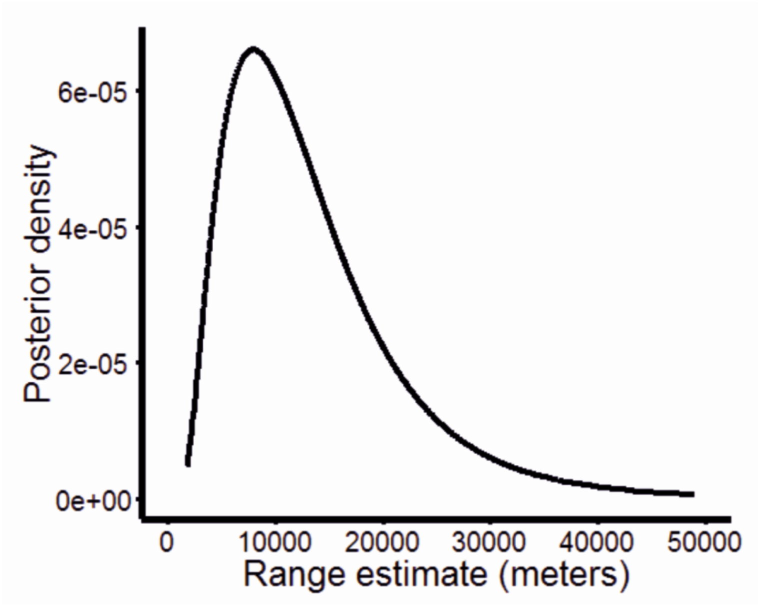

Fig. S4. Posterior distribution of the spatial range estimate for the fraction of infected leaves with resting structures for the pathogen *Podosphaera plantaginis* in its host populations in Åland, southwestern Finland. Shown are the results from year-specific spatial Bayesian models using LAPLACE approximation. The spatial pattern of resting spore production is visualized in Fig. 4 in the main manuscript.

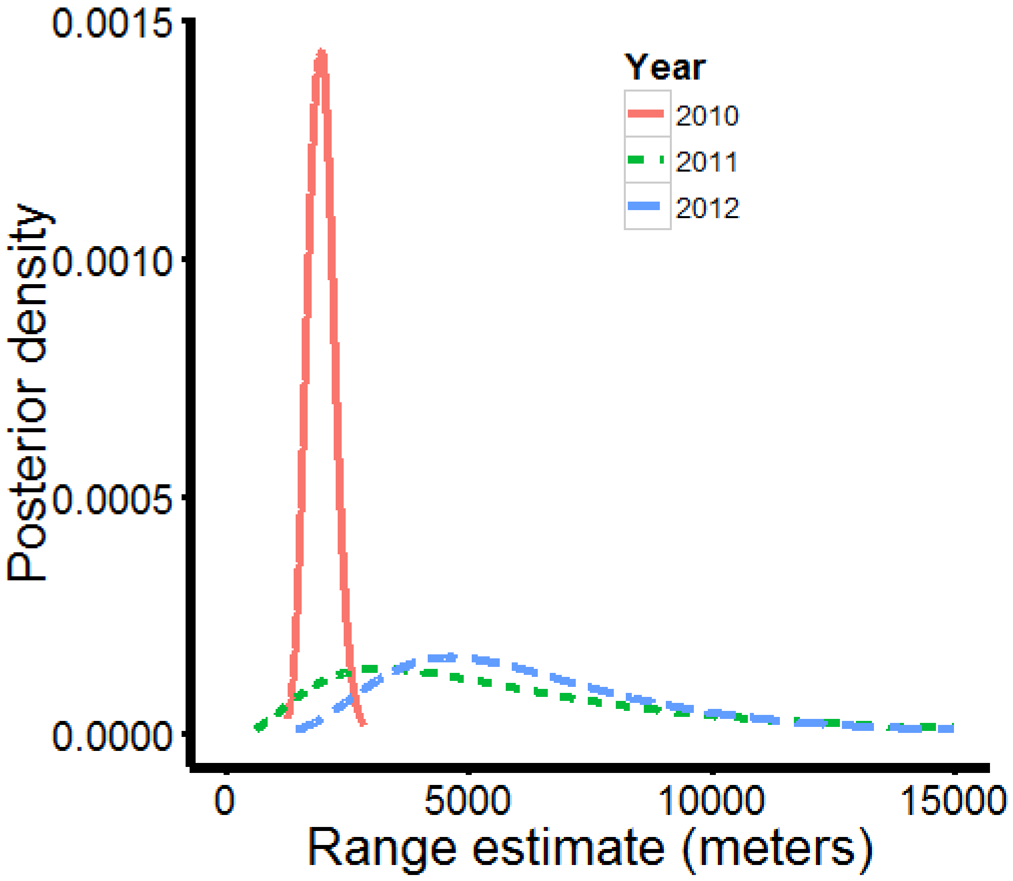

Fig. S5. Fraction of infected cage plants in the overwintering trial experiment. Leaves were stored indoors under multiple controlled temperatures (-10°C, -5.5°C, 0.1°C, 5°C, 10°C and field°C; the latter reflected the average monthly temperature during winter in Åland from October to April: 8.8°C, 4.0°C, 1.9°C, 2.0°C, 2.0°C, 0.8°C, and 4.7°C). The eight outdoor locations (populations 1043, 1413, 490, 1290, 4450, 876, 4698 and 8538) were distributed across Åland (squares in Fig. S2).

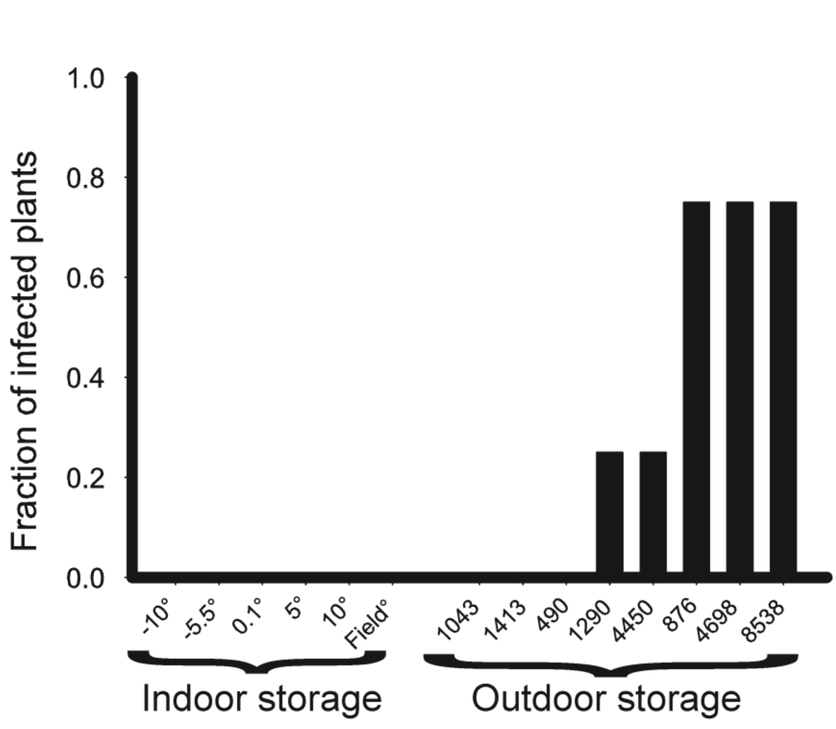

Notes S1. A trial experiment on overwintering survival using indoor and outdoor overwintering sites

Aims

We carried out a trial overwintering experiment to simultaneously i) provide a first demonstration that resting structures are able to infect plants in spring in this pathosystem, and ii) assess the impact of winter conditions on the survival of resting structures collected from different populations.

Materials and methods

We haphazardly collected leaves bearing resting structures in autumn 2010 from several populations (triangles and squares in Fig. S2). As a single leaf and population can contain multiple pathogen genotypes (Tollenaere *et al.*, 2012), we note that such an experiment cannot measure variation among individual pathogen genotypes for overwintering, but it may indicate whether there is differentiation among pathogen populations. We stored leaves from three populations (red triangles in Fig. S2) under multiple controlled indoor conditions (-10°C, -5.5°C, 0.1°C, 5°C, 10°C and 'field temperature'; the latter reflected the average monthly temperature during winter in Åland from October to April: 8.8°C, 4.0°C, 1.9°C, 2.0°C, 2.0°C, 0.8°C, and 4.7°C). Leaves from an additional four populations (green squares in Fig. S2) were stored reciprocally in each of the four populations from which the leaves were collected, as well as in an additional four populations (purple squares in Fig. S2). Leaves were stored individually in polyester pollination bags (PBS International) within the aforementioned indoor and field locations. In April 2011, leaves were taken from the indoor storages and field locations. To assess whether leaves bearing resting structures were able to infect plants in spring, we then hung two leaves bearing resting structures from the same 'population of origin / overwintering site' combination above a single plant individual using two vertical sticks and horizontal iron wire. Plants were individually caged using a polyester pollination bag (PBS International 10-1; 1-window; 255 x 510 mm), as previous work has shown that infection develops well in these bags, and spores cannot leave or enter (Laine, 2011). Plants were scored on 21 June for the presence of powdery mildew infection.

Results

Environmental conditions during the off-season had a major impact on viability and spring germination of the resting structures. The experiment revealed that none of the resting structures stored indoors were able to infect caged plants in spring, even though they were stored at a wide range of temperatures (-10 to +10 °C; Fig. S5). This is in striking contrast with an average infection

percentage of 34% (11 out of 32 caged plants) when resting structures were stored overwinter in natural populations (Fig. S5). There is also an indication that off-season survival in the field varied among the overwintering sites (Fig. S5).

Methods S1. A detailed description of the statistical methods.

To analyse the impact of environmental and spatial factors on the spatial pattern of winter extinction, July abundance and the proportion of infected leaves with resting structures, we fitted a Bayesian spatial model using the integrated nested Laplace approximation (Cameletti *et al.*, 2012) as implemented in the package *INLA* (Rue *et al.*, 2009; Lindgren *et al.*, 2011) in *R* version 2.15.1 (R Core Team, 2012). The advantage of this method is that it efficiently and accurately estimates both covariates and the spatial range of autocorrelation (as based on Euclidean distance between populations). For both overwintering survival and resting structure formation, we included the environmental variables distance to shore, plant dryness, patch shadow, habitat openness, July rainfall, August rainfall and population age (i.e. how many years ago the pathogen population had been established by colonization, with a maximum value of 5) and the spatial factors host plant coverage, road presence and host plant spatial connectivity as explanatory covariates. The average rainfall in July and August was estimated separately for each population using detailed radar-measured rainfall data. To reduce the number of covariates in the model we pre-selected the covariates to be included using a linear / logistic model and the function *stepAIC* with the option *'backwards'* (package *MASS*). Significance of the explanatory variables was then assessed based on the deviance information criterion (DIC) in the spatial Bayesian model.

To analyse the experimental data, we used the framework of generalized linear mixed-effects models (Littell *et al.*, 2006). All models were fitted with procedure GLIMMIX in SAS 9.3. For binomial data, we assumed a binomial distribution with a logit link. For models with multiple interactions, we used the principle of backwards stepwise model simplification to arrive at a minimum adequate model, where variables were retained when $p<0.1$ (Crawley, 2007). Significance for fixed and random effects was assessed using F-tests and log-likelihood ratio tests, respectively (Littell *et al.*, 2006).

In the overwintering experiment we aimed to investigate the impact of pathogen population of origin and overwintering site on overwintering success. To analyse this experiment, we modelled the response variables *Infection* (0/1) and *Proportion of infected leaves* (number of infected / total leaves) as a function of the fixed variable '*Number of resting structures*' (representing the impact of resting structure quantity) and the random variables '*Pathogen population of origin'*, '*Overwintering site*', and their interaction. We further added the variable *Micro-site* (nested within '*Overwintering site*') to account for micro-environmental variation during the off-season and '*Plant individual*' (nested within '*Pathogen population of origin'*) to account for variation among pathogens collected from different plants within the same population. Finally, we added the variable '*Receiving plant genotype*' to represent the impact of receiving host plant genotype during infection

in spring. When the interaction between '*Pathogen population of origin*' and '*Overwintering site'* was significant (as it was for disease intensity, see *Results*), we probed for a global pattern of local adaptation using two alternative models. In model 1, we constructed a similar model as above, where we specified all factors as fixed variables. We then used a least-squares means contrast to test for global local adaptation (by contrasting least-squares means for sympatric [local] and allopatric [non-local] pathogen population of origin / overwintering site combinations). In model 2, we modified the original model by including the fixed variable '*Sympatry'* (and removing the interaction term between '*Overwintering site*' and '*Population of origin*'). The term '*Sympatry'* (0/1) would capture variation in disease intensity resulting from infection by resting structures that were stored in sympatry or allopatry (i.e. resting structures that were overwintered in the location from which they were collected or in a non-local location, respectively).

Finally, we investigated the relationship between the presence of resting structures and off-season survival at two spatial scales. At the plant level, infection (0/1) and disease intensity (number of infected leaves / total number of leaves) in spring were modelled as a function of the number of leaves with resting structures. The pathogen population was used as random factor to account for variation among populations in overwintering success. At the patch level, off-season survival and July abundance were modelled as a function of the fraction of infected leaves with resting structures in the previous autumn.
